# Diagnosis of oesophageal cancer by detection of minichromosome maintenance 5 protein in gastric aspirates

**DOI:** 10.1038/sj.bjc.6602028

**Published:** 2004-07-20

**Authors:** G H Williams, R Swinn, A T Prevost, P de Clive-Lowe, I Halsall, J J Going, C N Hales, K Stoeber, S J Middleton

**Affiliations:** 1 Wolfson Institute for Biomedical Research and Department of Histopathology, University College London, The Cruciform Building, Gower Street, London WC1E 6BT, UK; 2 Department of Clinical Biochemistry, University of Cambridge, Level 4, Laboratory Block, Box 232, Addenbrooke's Hospital, Hills Road, Cambridge CB2 2QR, UK; 3 Department of Public Health and Primary Care, Centre for Applied Medical Statistics, University of Cambridge, University Forvie Site, Robinson Way, Cambridge CB2 2SR, UK; 4 Department of Urology, Box 43, Addenbrooke's Hospital National Health Service Trust, Hills Road, Cambridge CB2 2QQ, UK; 5 Department of Pathology, Glasgow University, Glasgow Royal Infirmary, Castle Street, Glasgow G4 OSF, UK; 6 Department of Gastroenterology, Addenbrooke's Hospital National Health Service Trust, Hills Road, Cambridge CB2 2QQ, UK

**Keywords:** Mcm5, DNA replication licensing, diagnosis, oesophageal cancer

## Abstract

Symptomatic oesophageal cancer is usually advanced and the prognosis poor. Lethality of symptomatic oesophageal cancer has motivated screening for these diseases earlier in their evolution, but reliable methods for early diagnosis remain elusive. We have demonstrated that dysregulated expression of minichromosome maintenance (MCM) proteins 2–7 is characteristic of early epithelial carcinogenesis, and that these key DNA replication initiation factors can be used as diagnostic markers for cervical and genito-urinary tract cancer. In this study, we investigated whether minichromosome maintenance protein 5 (Mcm5) can be used to detect oesophageal cancer cells in gastric aspirates. Two monoclonal antibodies raised against His-tagged human Mcm5 were used in a time-resolved immunofluorometric assay to measure Mcm5 levels in cells isolated from gastric aspirates of 40 patients undergoing gastroscopy for suspected or known oesophageal carcinoma or symptoms of dyspepsia. The test discriminated with high specificity and sensitivity between patients with and without oesophageal cancer (85% sensitivity (95% confidence interval (CI)=62–97%), 85% specificity (CI=66–96%)), as demonstrated by the large area under the receiver operating characteristics curve (0.93 (95% CI=0.85–0.99)). Elevated levels of Mcm5 in gastric aspirates are highly predictive of oesophageal cancer. This simple test for oesophageal cancer is readily automated with potential applications in primary diagnosis, surveillance and screening.

The incidence of oesophageal cancer in Western societies is increasing rapidly (Blot *et al*, 1991) and currently stands at approximately 6 per 100 000 in England and Wales (Office for National Statistics England and Wales, 1999). It accounts for about 6700 cancer-related deaths per year in the UK (Cancer Research Campaign, 1998, 1999). Most patients are not candidates for curative treatment as the symptoms associated with oesophageal cancer predominantly arise when the tumour is at an advanced stage. Without curative treatment, the overall mean survival of patients with oesophageal cancer is 6 months, even including those patients deemed suitable for curative treatment, the overall survival is poor, about 5% at 5 years (Newnham *et al*, 2003).

However, when applied to patients with very early disease, the latest chemotherapy regimes offer excellent results, and 5-year survival for T1–2N0 disease is now greater than 80% (Urschel and Vasan, 2003). Therefore, at present, the key to successful treatment of oesophageal cancer is early diagnosis and there is consequently great interest in the development of a screening test that will identify patients with asymptomatic oesophageal malignant or premalignant disease. Patients with Barrett's oesophagus (Miros *et al*, 1991), known to have a high risk of developing oesophageal carcinoma, can be screened endoscopically, but this is time consuming and of questionable effectiveness. An extension of current endoscopic screening to include patients with gastro-oesophageal reflux disease would have even less cost effectiveness, even though this group is at increased risk of developing oesophageal cancer (Iftikhar *et al*, 1992). Certain risk factors such as cigarette smoking (Brown *et al*, 1994; Hu *et al*, 1994; Rolon *et al*, 1995), alcohol (Brown *et al*, 1994; Hu *et al*, 1994; Rolon *et al*, 1995) and age (Office for National Statistics England and Wales, 1999) are also associated with higher risk but again lack suitable specificity to be useful as screening tools.

Population screening for squamous oesophageal carcinoma is only practised where the incidence is high such as Japan and China using techniques such as abrasive cytology, barium oesophagography and fibreoptic oesophagoscopy (Riddell, 1996). This is a labour-intensive approach requiring skilled cytopathologists, gastroenterologists and radiologists. Dysplasia and cancer surveillance by endoscopy and biopsy of Barrett's patients in Western populations is also undertaken, but with uncertain benefit (Macdonald *et al*, 2000).

Despite advances in the molecular pathology of oesophageal neoplasia, no useful clinical biomarkers have yet been identified for diagnostic and screening applications. The initiation of DNA replication represents a final and critical step in growth regulation and lies downstream at the convergence point of growth regulatory pathways (Williams and Stoeber, 1999). Proteins of the minichromosome maintenance (MCM) family (minichromosome maintenance protein 2–7, Mcm2–7) play a critical role (DNA replicative helicase) in the initiation of DNA replication (Bell and Dutta, 2002). We have previously demonstrated that dysregulation of MCM proteins is an early event in epithelial carcinogenesis, resulting in exfoliation of MCM-positive tumour cells, and have used these novel biomarkers of growth in diagnostic screening applications for cervical and genitourinary tract cancer (Williams *et al*, 1998; Stoeber *et al*, 1999; Stoeber *et al*, 2002). Moreover, we have shown that dysregulation of the DNA replication initiation pathway is an early event in oesophageal carcinogenesis with aberrant expression of MCM proteins occurring in both squamous dysplasia and glandular dysplasia complicating Barrett's oesophagus (Going *et al*, 2002).

These data suggest that detection of MCM proteins in exfoliated tumour cells might provide a potentially sensitive indicator of oesophageal neoplasia. Here, we describe an evaluation of this approach using a liquid phase immunofluorometric assay to measure quantitatively Mcm5 levels in gastric luminal samples obtained from patients undergoing gastroscopy for upper gastrointestinal symptoms. Gastric luminal secretions can be obtained without the need for endoscopy, and can be collected in health centres as a screening tool.

## MATERIALS AND METHODS

### Study subjects

Gastric aspirates were obtained from 40 patients undergoing gastroscopy at Addenbrookes Hospital National Health Service Trust (Cambridge, UK) for suspected or known oesophageal carcinoma or symptoms of dyspepsia. All patients gave full consent and the study was approved by the Local Research Ethics Committee. Aspirates were obtained through the endoscope suction channel. A full endoscopic examination of the oesophagus, stomach and duodenum was performed. Endoscopies were undertaken with conscious sedation using Midazolam. Punch biopsies were taken from regions that looked abnormal to identify any underlying pathological process. Aspirates were analysed in a blinded manner for immunofluorometric Mcm5 detection. On completion of the study, patient data were decoded and the immunofluorometric signals compared with endoscopy and biopsy histology results.

### Gastric aspirates collection and storage

Prior to biopsy sampling, an endoscope was passed carefully down the oesophagus, which was inflated with air thus minimising any contact with the oesophageal surface. Gastric juice was then aspirated immediately to reduce instrument-related trauma. Gastric aspirates were kept on ice until processing for storage. Storage buffer (10 × phosphate-buffered saline (PBS), 5% bovine serum albumin (BSA), 1 M sucrose, 0.2% NaN_3_) containing one complete EDTA-free protease inhibitor cocktail tablet (Roche Diagnostics Ltd, Lewes, East Sussex, UK) per 10 ml of buffer was added to gastric aspirates at one-tenth aspirate volume and carefully mixed with the sample. Gastric aspirates in storage buffer were transferred into 5 ml cryovials and stored in liquid nitrogen (LN_2_) for cryopreservation. The aspirates were stored in LN_2_ within 5 h of the samples being collected.

### Processing of standards and gastric aspirates

Standards for the immunofluorometric Mcm5 assay were prepared, and standards and gastric samples processed as described previously (Stoeber *et al*, 2002). Briefly, standards and clinical samples were thawed, and the cells were isolated by centrifugation at 1500 *g* for 5 min at 4°C. The supernatants were discarded, and the cell pellets were washed three times with 500 *μ*l of PBS. Cell pellets were resuspended in 250 *μ*l (for those pellets with a volume less than approximately 200 *μ*l) or 500 *μ*l (for those pellets with a volume greater than approximately 200 *μ*l) of processing buffer (PBS, 0.4% sodium dodecyl sulphate (SDS), 0.02% NaN_3_). Cell lysates were prepared by incubating the resuspended samples at 95°C for 45 min. The DNA in each sample was sheared by passing the lysates through a 21-gauge needle (Becton Dickinson UK Ltd, Cowley, Oxford, UK), and nucleic acids were digested with DNase I (20 U ml^−1^; Roche Diagnostics) and RNase A (1 *μ*g ml^−1^; Roche Diagnostics) for 2 h at 37°C. The samples were centrifuged at 15 000 **g** for 10 min to pellet the cell debris, the supernatants were collected and 50 *μ*l of each was directly used in the immunofluorometric assay.

### Immunofluorometric measurement of Mcm5 levels in gastric aspirates

Monoclonal antibodies (MAbs) 12A7 and 4B4 raised against His-tagged human Mcm5 were protein A purified from hybridoma supernatants as described previously (Stoeber *et al*, 2002). Protein A-purified MAb 4B4 was labelled with europium using a DELFIA® Eu-labelling kit (Perkin-Elmer Life Science, Wallac Oy, Turku, Finland) according to the manufacturer's instructions. The assay was standardised using HeLa cells as described previously (Stoeber *et al*, 2002), and one fluorescence unit was defined as the signal generated by the Mcm5 contents of one proliferating HeLa S3 cell, approximately 10^5^ Mcm5 molecules (Kearsey and Labib, 1998). DELFIA® research reagents were obtained from Perkin-Elmer Life Science. All other reagents were obtained from Sigma-Aldrich.

Immunofluorometric measurements of Mcm5 levels were performed as described previously (Stoeber *et al*, 2002). Standard curves were constructed from fluorescence values generated by the blank and standard wells, and the fluorescence values of the gastric aspirate samples were calculated with the Multicalc Advanced Immunoassay Data Management package (Perkin-Elmer Life Science). For immunofluorometric measurement of Mcm5 levels, assay standards, control samples and gastric aspirate samples were run as duplicates and the mean of the duplicate results reported. For acceptance of immunofluorometric measurements in the assay, the following coefficients of variations were required: CV <20% for results between 1500 and 5000 cells well^−1^ standard curve points; CV <15% for results between 5000 and 15 000 cells well^−1^; and CV <10% for results >15 000 cells well^−1^.

### Immunoassay performance

In our analysis, we used 1500 cells well^−1^ as the lower detection limit because the within-batch coefficient of variation of the assay was less than 25% in all samples with cell dilutions above 1500 cells well^−1^, but in only one-quarter of samples below this limit. Samples that generated a fluorescence signal below that corresponding to 1500 cells well^−1^ were reported as having fewer than 1500 cells well^−1^.

### Immunohistochemistry

Formalin-fixed, paraffin-embedded surgical biopsy material from tumour-positive cases was selected for immunohistochemical analysis. Automatic immunostaining for Mcm2 and Mcm5 was performed on a DAKO Techmate™ 500 as described previously (Stoeber *et al*, 2001). Primary antibodies were omitted in negative controls and in addition appropriate tissue sections were used as positive and negative controls. Microscopic images were acquired with an Olympus BX51 light microscope/CCD camera set-up and ANAlysis image capturing software (Soft Imaging Systems GmbH, Münster, Germany). A semi-quantitative determination of the extent of staining was obtained by calculating a labelling index for each protein stained. At least 200 nuclei were assessed per case. Results were expressed as a percentage of positively stained nuclei out of the total number of nuclei counted in representative microscopic fields. The median and range of labelling indices were calculated.

### Statistical analysis

Sensitivity and specificity characteristics of the immunofluorometric Mcm5 test for the detection of oesophageal cancer are presented as a receiver operating characteristics (ROC) curve. The area under the nonparametric ROC curve was used to assess the overall accuracy of the test (McNeil and Hanley, 1984; Altman and Bland, 1994). Three cut points were used to demonstrate test performance under different circumstances as follows: at the lower detection limit of the assay (i.e. 1500 cells well^−1^), where sensitivity of the test was maximal; at the point where the false-positive and false-negative rates of the test were equal (i.e. 5000 cells well^−1^); and where specificity exceeded 95% (i.e. 7500 cells well^−1^). An exact 95% confidence interval (CI) for each proportion, including sensitivity, specificity and predictive values of Mcm5 and cytology, was derived assuming a binomial distribution using StatXact software, Version 4.0 (Cytel Software Corporation, Cambridge, MA, USA). Unless otherwise stated, statistical tests were performed using SPSS software, Version 11.5 (SPSS Inc., Chicago, IL, USA). The level of the signal was compared between patient groups using the Kruskal–Wallis test and for pairs of groups using the Mann–Whitney *U*-test. All statistical tests were two-tailed, and a 5% level was used to indicate statistical significance.

## RESULTS

The patient characteristics, clinical symptoms on presentation, endoscopy findings and histopathological diagnoses derived from the 47 gastric aspirate samples were obtained for analysis (Table 1

**Table 1:** Patient demographics and sample characteristics at endoscopy and biopsy

**Patient characteristics (*n*=40 patients)**	***N* (%) or median (interquartile range)**
*Sex*	
Male	24 (60%)
Female	16 (40%)
Age (years)	74 (58–82)
	
*Sample characteristics (n*=*47 samples)*^a^	
Gastric aspirate volume (ml)	4 (4–4.5)
	
*Endoscopy and/or biopsy findings*	
Tumour absent	27 (57%)
Normal oesophagus	2
Diverticulum	1
Shatski ring	1
Chemical gastropathy	1
Oesophagitis^b^	9
Barrett's oesophagus without dysplasia	13
	
*Tumour present*	20 (43%)
AdCa^c^	10
SCC	10
	
*Tumour stage*	
T2N0M0	1
T3N0M0	9
T3N0M1	1
T3N1M0	1
T3N1M1	1
T4N1M0	1

AdCa=adenocarcinoma; SCC=squamous cell carcinoma.

a Two samples from one patient with AdCa, four samples from one patient and two samples from two patients with SCC, and two samples from one patient with Barrett's oesophagus with associated inflammation and ulceration were included in the analysis.

b Seven patients with oesophagitis had ulceration.

c Three AdCa's had associated severely atypical Barrett's oesophagitis.

). Five of the 40 patients provided more than one sample during follow-up. The patients’ median age was 74 years (range 40–90 years) and 60% were male. The 20 (43%) tumour samples comprised 10 adenocarcinomas (AdCa) and 10 squamous cell carcinomas (SCC). Three of the AdCas were found in association with severely dysplastic Barrett's oesophagus. A total of 13 patients were diagnosed with metaplastic Barrett's oesophagus without associated dysplasia. Seven patients were found to have benign ulceration in a background of inflammatory oesophagitis.

**Figure 1:**
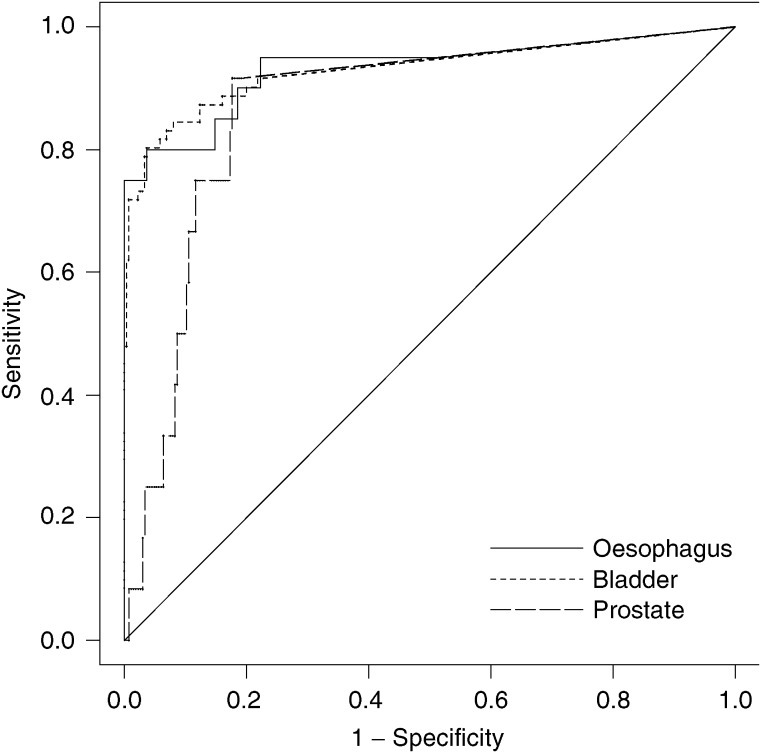
ROC curve of immunofluorometric Mcm5 test. The jagged curve (solid line) is the nonparametric ROC curve. The diagonal line is a reference line. Area under curve=0.93 (95% CI=0.85–0.99). ROC curves for detection of bladder cancer (jagged dotted line) and prostate cancer (jagged dashed line) are shown for comparison.

The performance of the immunofluorometric Mcm5 assay as a diagnostic test for oesophageal cancer is shown as a ROC curve (Figure 1

). The test discriminated, with high specificity and sensitivity, between patients with and without oesophageal cancer, as demonstrated by the large area under the ROC curve (0.93 (95% CI=0.85–0.99)), which was statistically significantly larger than the area predicted by the null hypothesis (0.5) (*P*<0.001). In other words, a randomly selected patient with oesophageal cancer would have a 93% probability of having an immunofluorometric Mcm5 value that is larger than a randomly selected patient without a malignancy.

Evaluation of the test is demonstrated in Table 2

**Table 2:** Sensitivity, specificity and predictive values of the Mcm5 test for oesophageal cancer

**Test**	**% Sensitivity (95% CI)**	**% Specificity (95% CI)**	**% PPV (95% CI)**	**% NPV (95% CI)**
Mcm5 test ⩾1500 cut point	95 (75–99)	48 (29–68)	58 (39–75)	93 (66–99)
Mcm5 test ⩾5000 cut point	85 (62–97)	85 (66–96)	81 (58–95)	88 (70–98)
Mcm5 test ⩾7500 cut point	75 (51–91)	96 (81–99)	94 (70–99)	84 (66–95)

Mcm5=minichromosome maintenance protein 5; CI=confidence interval; PPV=positive predictive value; NPV=negative predictive value.

a Three cut-off points were used to demonstrate test performance under different circumstances as follows: at the lower detection limit of the assay (fluorescence signal generated from 1500 proliferating HeLa S3 cells well^−1^), where sensitivity of the test was maximal; at the point where the false-positive and false-negative rates of the test were equal (5000 cells well^−1^); and where specificity exceeded 95% (7500 cells well^−1^).

at different performance levels at cut-point values of 1500 cells well^−1^ (lower detection limit of the assay), 5000 cells well^−1^ (equal false-positive and false-negative rates) and 7500 cells well^−1^ (high specificity). At the 1500-cell cut point, the test had 95% (19 of 20) sensitivity and 58% (19 of 33) positive predictive value. At the 5000-cell cut point, the test had 85% (17 of 20) sensitivity and 81% (17 of 21) positive predictive value. At the 7500-cell cut point, the test had 75% (15 of 20) sensitivity and 94% (15 of 16) positive predictive value.

Table 3

**Table 3:** Immunofluorometric Mcm5 test performance in patient groups

**Patient groups and subgroups**	**Group size (*N*)**	**Median signal**	**Interquartile range**	**% Signals ⩾5000 (95% CI)**
Negative for cancer	27	1718	<1500–2897	15 (4–34)
No ulceration	20	<1500	<1500–<2115	5 (1–25)
Ulceration	7	4871	2897–6732	43 (10–82)
Oesophageal cancer	20	16 401	7263–29 822	85 (62–97)
AdCa	10	11 210	5023–23 692	80 (44–97)
SCC	10	25 036	9752–34 221	90 (56–99)

Mcm5=minichromosome maintenance protein 5; CI=confidence interval; AdCa=adenocarcinoma; SCC=squamous cell carcinoma.

and Figure 2

**Figure 2:**
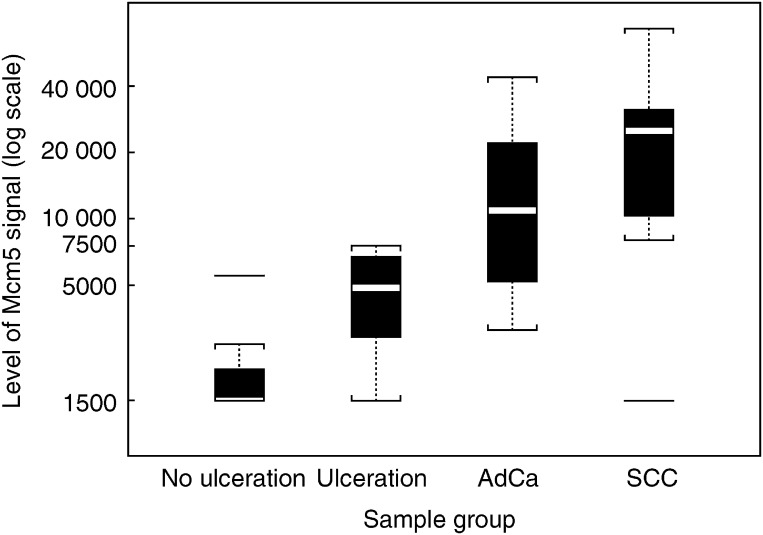
Mcm5 signal by sample group. Each box represents the interquartile range of the Mcm5 signal data for the corresponding sample group. The horizontal line inside the box represents the median signal. Any signal further than 1.5 times the interquartile range is considered as an outlying signal value and plotted separately. The dotted lines extend 1.5 times the interquartile range from the limits of the box.

show the performance of the immunofluorometric Mcm5 test according to the diagnosis made at clinical follow-up using endoscopy findings and histopathological diagnosis as the gold standard. The level of signal in the four subgroups was significantly different (Kruskal–Wallis test, *P*<0.001). Interestingly, the Mcm5 immunofluorometric signal for patients with ulceration (median 4871) was higher than for other patients without malignancy, where the median signal was below the lower detection limit of 1500 cells well^−1^ (Mann–Whitney *U*-test, *P*=0.002). The level of signal was not significantly different (Mann–Whitney *U*-test, *P*=0.16) between AdCas (median 11 210) and SCCs (median 25 036). The largest difference (Mann–Whitney *U*-test, *P*<0.001) was between those samples from patients without tumour (median 1718) and those with tumour (median 16 401).

**Figure 3:**
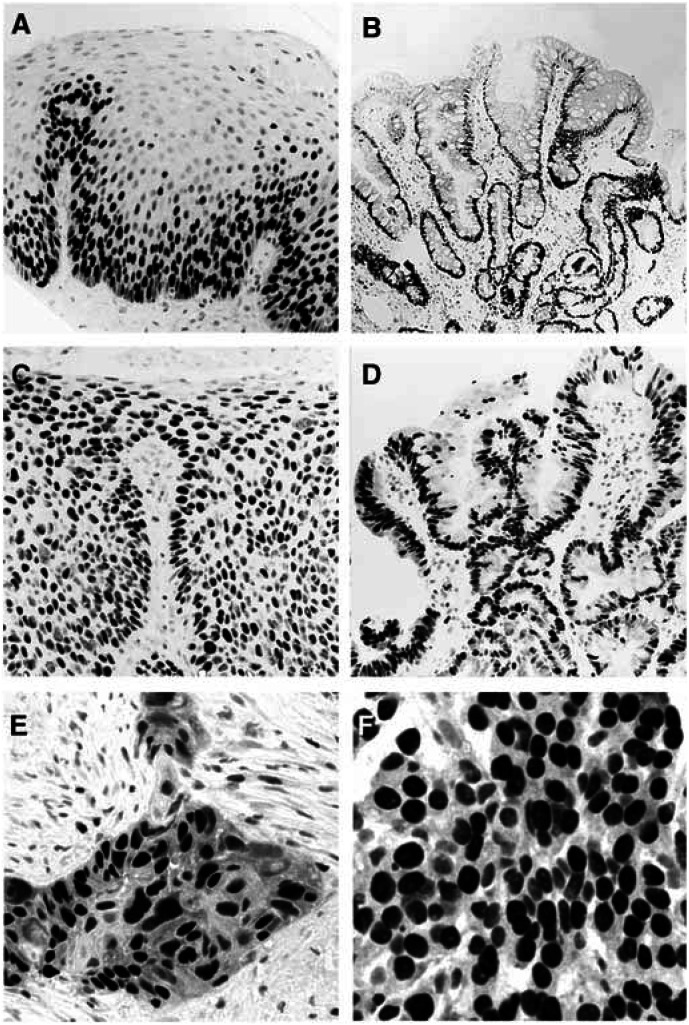
Mcm2 protein expression in normal and neoplastic oesophagus. (**A**) Normal squamous epithelium showing Mcm2 expression restricted to the basal proliferative compartment. Onset of the differentiation programme is associated with downregulation of the MCM replication licensing factors. Mcm2 expression is undetectable in surface terminally differentiated cells. (**B**) Nondysplastic testinal-type ‘specialised’ Barrett's mucosa showing Mcm2 expression in cells in the proliferative zone beneath the mucosal surface. Mcm2 expression is markedly downregulated as cells execute their differentiation programme and migrate onto the mucosal surface. (**C**) Low- to high-grade squamous dysplasia showing high levels of Mcm2 expression. The arrest in differentiation is associated with persistant Mcm2 expression in surface layers. (**D**) Low-grade Barrett's dysplasia showing high levels of Mcm2 expression in upper crypts and surface layers. The failure of dysplastic cells to execute their differentiation programme (maturation arrest) is associated with persistent expression of the MCM replication licensing factors in upper crypt and surface epithelium. (**E**) Moderately to poorly differentiated SCC showing high levels of Mcm2 expression. Occasional viable Mcm2 negative cells are present showing morphological features of differentiation (keratinisation). (**F**) Poorly differentiated AdCa showing high levels of Mcm2 expression. Occasional viable Mcm2-negative cells are also present showing some features of glandular differentiation (mucin production).

The elevated levels of the Mcm5 DNA replication licensing protein found in gastric aspirates from patients with oesophageal cancer is consistent with our previous immunohistochemical findings demonstrating aberrant expression of Mcm2 and Mcm5 proteins in dysplastic and malignant oesophageal lesions (Going *et al*, 2002). Immunohistochemical analysis of the tumours detected in this study confirms our previous findings showing high levels of MCM protein expression, with the majority of tumour cells expressing the Mcm2 and Mcm5 replication licensing factors (SCCs: Mcm2 (92–98%, mean: 95%), Mcm5 (93–100%, mean: 96%); AdCas: Mcm2 (83–99%, mean: 94%), Mcm5 (90–98%, mean: 94%); Figure 3

).

## DISCUSSION

Patients with oesophageal cancer present at an advanced stage, symptoms are usually of recent origin and their period of survival is short. The incidence of oesophageal cancer is increasing and therefore there is an urgent need for reliable cost effective methods for early diagnosis (Wang *et al*, 1997). Abrasive brush cytology as a screening technique for oesophageal cancer has been used for many years in high incidence areas of China (Shu, 1983). Although the brush biopsy capsule is inexpensive, the hidden costs including preparation of slides and expert cytopathological assessment are considerable. Moreover, despite the alarming increase in the incidence of AdCa of the oesophagus in North America and Europe, screening for neoplasia in Barrett's oesophagus is controversial partly due to the invasive nature and expense of introducing endoscopic biopsy surveillance programmes (Wright *et al*, 1996).

Maturation arrest and failure to engage correctly the differentiation programme, the hallmark of dysplastic precancerous lesions, is associated with aberrant expression of the MCM replication initiation factors (Williams *et al*, 1998; Stoeber *et al*, 1999; Going *et al*, 2002; Stoeber *et al*, 2002). Importantly, aberrant expression of MCM proteins was identified in both squamous dysplasia and Barrett's glandular dysplasia, but not in Barrett's metaplastic oesophagus (Figure 3; Going *et al*, 2002). We have previously shown that detection of MCM proteins in urine sediments is a sensitive and specific test for urothelial neoplasia allowing detection of bladder cancers at all stages and grades including severe dysplasia/carcinoma *in situ*, the latter corresponding to a similar step in tumour progression represented by dysplastic Barrett's oesophagus (Stoeber *et al*, 1999, 2002).

Using a similar analytical approach previously applied to the genitourinary tract (Stoeber *et al*, 2002), application of the immunofluorometric Mcm5 test to gastric aspirates has resulted in a strikingly similar performance (Tables 2 and 3 and Figure 1). Patients with tumours, including three cases with associated severe dysplastic Barrett's oesophagus, were detected with high sensitivity (85–95% at the low cut-off point). Importantly, inflammatory conditions including oesophagitis and Barrett's metaplastic oesophagus were not associated with false-positive results. Interestingly, ulcerative lesions gave a signal, but with an amplitude below that generated by tumours, most likely reflecting reparative growth with exposure of the stem-transit compartment to luminal secretions and the shedding of reactive Mcm5-positive cells. Similar results were found in the urinary tract in relation to renal calculi (Stoeber *et al*, 2002).

The immunofluorometric Mcm5 test provides a new approach to the detection of oesophageal cancer. Given the magnitude in the difference between Mcm5 levels in benign and malignant disease found in this study, it is likely that even small cases at an early stage will be detected. Studies on large unselected populations will now be required to determine whether this novel diagnostic approach can be exploited as a screening tool to detect early curable tumours. Furthermore, our previous studies have shown that aberrant expression of the MCM proteins is a powerful marker of dysplasia in Barrett's oesopagus (Going *et al*, 2002). These data suggest that the immunofluorometric Mcm5 test could be further refined for screening of Barrett's oesophagus by employing balloon cytology catheters, increasing the yield of cellular material for biochemical analysis (Liu *et al*, 1994; Sepehr *et al*, 2000). The sensitivity and specificity data are strikingly similar when comparing oesophageal cancer with genitourinary tract cancers. The areas under the ROC curve for oesophagus, bladder and prostate are all around 0.93 (Figure 1; Stoeber *et al*, 2002). It is likely that similar results will be obtained for other cancers arising from self-renewing tissues using this approach.

These pilot data, including our previous studies examining the expression profile of MCM proteins during oesophageal carcinogenesis, suggest that immunofluorometric detection of Mcm5 in gastric aspirates provides a new approach for the detection of oesophageal neoplasia. Its use as a screening and diagnostic tool for oesophageal neoplasia needs to be urgently investigated considering the increasing incidence and high mortality rate associated with this disease.
